# Intracellular Water Exchange for Measuring the Dry Mass, Water Mass and Changes in Chemical Composition of Living Cells

**DOI:** 10.1371/journal.pone.0067590

**Published:** 2013-07-02

**Authors:** Francisco Feijó Delgado, Nathan Cermak, Vivian C. Hecht, Sungmin Son, Yingzhong Li, Scott M. Knudsen, Selim Olcum, John M. Higgins, Jianzhu Chen, William H. Grover, Scott R. Manalis

**Affiliations:** 1 Department of Biological Engineering, Massachusetts Institute of Technology, Cambridge, Massachusetts, United States of America; 2 Computational and Systems Biology Initiative, Massachusetts Institute of Technology, Cambridge, Massachusetts, United States of America; 3 Department of Mechanical Engineering, Massachusetts Institute of Technology, Cambridge, Massachusetts, United States of America; 4 Koch Institute for Integrative Cancer Research, Massachusetts Institute of Technology, Cambridge, Massachusetts, United States of America; 5 Center for Systems Biology, Massachusetts General Hospital, Boston, Massachusetts, United States of America; 6 Department of Pathology, Massachusetts General Hospital, Boston, Massachusetts, United States of America; 7 Department of Systems Biology, Harvard Medical School, Boston, Massachusetts, United States of America; 8 Department of Biology, Massachusetts Institute of Technology, Cambridge, Massachusetts, United States of America; Texas A&M University, United States of America

## Abstract

We present a method for direct non-optical quantification of dry mass, dry density and water mass of single living cells in suspension. Dry mass and dry density are obtained simultaneously by measuring a cell’s buoyant mass sequentially in an H_2_O-based fluid and a D_2_O-based fluid. Rapid exchange of intracellular H_2_O for D_2_O renders the cell’s water content neutrally buoyant in both measurements, and thus the paired measurements yield the mass and density of the cell’s dry material alone. Utilizing this same property of rapid water exchange, we also demonstrate the quantification of intracellular water mass. In a population of *E. coli*, we paired these measurements to estimate the percent dry weight by mass and volume. We then focused on cellular dry density – the average density of all cellular biomolecules, weighted by their relative abundances. Given that densities vary across biomolecule types (RNA, DNA, protein), we investigated whether we could detect changes in biomolecular composition in bacteria, fungi, and mammalian cells. In *E. coli*, and *S. cerevisiae*, dry density increases from stationary to exponential phase, consistent with previously known increases in the RNA/protein ratio from up-regulated ribosome production. For mammalian cells, changes in growth conditions cause substantial shifts in dry density, suggesting concurrent changes in the protein, nucleic acid and lipid content of the cell.

## Introduction

The dry and wet content of the cell as well as its overall chemical composition are tightly regulated in a wide range of cellular processes. Bacteria and yeast increase their ribosomal RNA content to achieve faster growth rates [1]–[4], the wet and dry content of yeast can change disproportionately during the cell cycle [5]–[7] and the water content of mammalian cells is reduced following apoptosis [8]. Despite the fundamental significance of these physical parameters, the techniques for measuring them directly, particularly in living cells, are limited. Dry and wet mass are typically obtained by weighing a population before and after baking to remove the intracellular water [9]. Although dry mass can be measured in living cells by quantitative phase microscopy [10], the conversion factor between refractive index and dry mass concentration must be known. While this factor is similar for most globular proteins (typically varying by less than 5%), it can vary by almost 20% for carbohydrates or lipids [10]. Approaches based on vibrational spectroscopy can provide chemical composition of living cells [11], but do not reveal the dry and wet mass.

**Figure 1:**
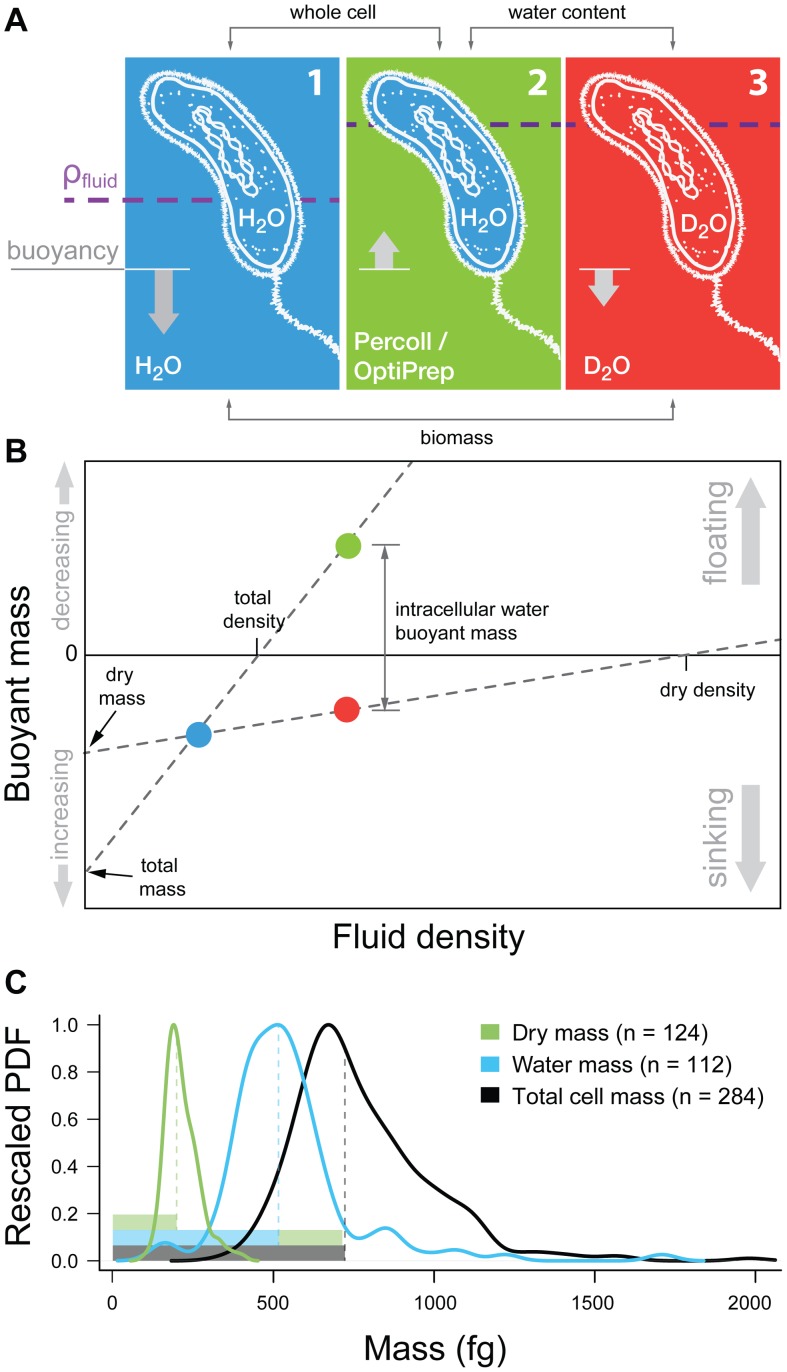
Buoyancy of a cell in fluids of different densities and membrane permeabilities. **a)** In an H_2_O or D_2_O based fluid (1 or 3), the cell sinks as a result of the dry content’s density being higher than the surrounding fluid. In a dense impermeable fluid (2), the buoyancy of the cell’s water content dominates and the cell floats. **b)** The pairing of the different buoyant mass measurements allows the determination of different biophysical parameters of the cell as shown in the plot (not to scale). **c)** Kernel density estimates of probability densities for dry mass, water mass and total mass of a sample of fixed stationary-phase *E. coli*. Functions were rescaled so that their maxima were one. Solid bars represent sample medians.

To address these limitations, we developed an approach that exploits the high water permeability of cellular membranes for obtaining the water mass, dry mass, and an index of chemical composition for living cells (**Fig. 1**). When a cell is weighed in fluids of distinct densities - an H_2_O-based and a deuterium oxide-based (D_2_O) fluid - the aqueous portion of the cell is neutrally buoyant in both measurements since intracellular H_2_O is rapidly replaced by D_2_O upon immersion in D_2_O. The paired weighings (**Fig. 1a,b**, blue and red) therefore offer direct quantification of the cell’s dry mass and its non-aqueous volume, which allows us to determine a parameter termed dry density [12], [13] – the density of the cell’s dry material (**Fig. 1b**). If we instead make the first measurement in an impermeable fluid as dense as D_2_O, the intracellular H_2_O buoys up the cell. Upon immersing the cell in D_2_O, the intracellular H_2_O is replaced by D_2_O, and the aqueous portion of the cell no longer contributes to its buoyancy. The differential between these two measurements (**Fig. 1a,b**, green and red) yields the intracellular water mass, as it excludes the dry material whose buoyant mass is identical in both cases.

Here we validate this approach and use it to measure the dry mass and dry density of various cell types, from microbes to mammalian cells. Dry density is related to the chemical composition of cells: it is an average of the densities of the different components of the cell's biomass (RNA, proteins, lipids, etc.) (**Table 1**) weighted by their relative amounts (**Table 2**). It is different from dry mass density, which refers to the concentration of cellular dry mass, i.e. dry mass per unit cell volume. In contrast to total cell density or dry mass density, dry density is independent of the cell's water content, making the measurement invariant to water uptake or expulsion due to osmotic pressures. Dry density is also size independent, whenever the relative chemical composition remains unchanged.

**Table 1:** Density of chemical components of cells.

	Density (g⋅cm^–3^)	References
**DNA**	1.4–2.0	[31], [32]
**RNA**	2.0	[31]
**Protein**	1.22–1.43	[31], [33]

**Table 2:** Approximate chemical composition of a bacterium, yeast and mammalian cell.

		*E. coli*		*S. cerevisiae*	Mammalian Cell
% total weight	**Water**		70	80	70
% dry weight	**DNA**		3	0.1–0.6	1
	**RNA**		20	6–12	4
	**Proteins**		50–55	35–60	60
	**Lipids**		7–9	4–10	13
	References		[15], [34], [35]	[36]–[40]	[35]

We show that dry density increases between stationary and exponential phases in *E. coli* and *S. cerevisiae,* as might have been expected due to known changes in RNA/protein ratio, since RNA is denser than most cellular components. We further observe changes in dry density of mammalian cells that are manifestations of their different states: healthy proliferating mouse embryonic fibroblasts, FL5.12 cells and L1210 lymphocytic leukemia cells all show higher dry density values than confluent fibroblasts, nutrient-starved FL5.12 cells and cycloheximide-treated L1210 cells, respectively, even though in some cases their dry mass distributions do not undergo noticeable alterations. These examples suggest that dry density may be used to determine the bulk cellular composition that is necessary for proliferation.

### Measurement Principle

This work builds upon a previously published method for measuring a particle’s total density, mass and volume. Like Grover et al. [14] we use a suspended microchannel resonator (SMR) to determine a single particle’s buoyant mass, defined as.
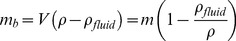
(1)where 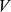
is the volume, *m* is the mass and 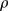 is the density of the particle immersed in a fluid of density 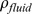 (**Fig. S1**). One buoyant mass measurement does not uniquely determine either the volume or the mass of a particle, but with two sequential buoyant mass measurements in fluids of differing densities, it is possible to solve for the particle’s mass and volume (**Fig. 1b**).

We alter this method by rendering the intracellular water content of a cell neutrally buoyant in both buoyant mass measurements, allowing the paired measurements to isolate the physical properties of the dry content alone. We formalize this by decomposing a cell’s buoyant mass into two parts – the buoyant mass of the dry material and the buoyant mass of the intracellular water:
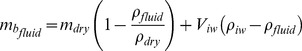
(2)


where 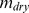 and 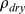 are the mass and density of the cell’s dry content, or biomass, and 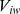, 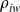 are the volume and the density of the exchangeable water content. Assuming that the cell is measured first in pure H_2_O and secondly in pure D_2_O, and that the intracellular H_2_O molecules are all replaced by D_2_O molecules, in each measurement the buoyant mass of the exchanged volume (the latter term in **equation 2**) is zero. The two cases yield.
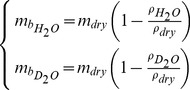
(3)and we can solve for the dry mass, dry volume, and dry density (**Fig. 1b**).

Additionally, the method can be easily modified to determine the cell’s water content, owing to the rapid exchange of H_2_O by D_2_O. A cell is first weighed in a dense, non-cell permeable fluid such as OptiPrep (iodixanol in H_2_O) and then weighed in D_2_O. If the fluids’ densities are adjusted to match, the contribution to the cell’s buoyant mass of the dry content (first term in **equation 2**) is identical in both fluids. Therefore the differential measurement allows for the determination of the mass and volume of the cell’s water content, since the value is simply the buoyant mass of the intracellular water when weighed in the non-cell permeable fluid. Further analysis of the method and assumptions is in the **Supporting Information**.

## Results

### Aqueous, Non-aqueous and Total Cellular Content

As an initial test of our method, we separately determined the water content, dry content and total content of individual cells from a sample of early stationary *E. coli*. Since the measurement time typically exceeded several doublings of the culture, cells were fixed to ensure all cells were representative of the culture at a single timepoint. We measured the single-cell water mass distribution by sequentially measuring the cells in OptiPrep:PBS (ρ = 1.101 g⋅cm^–3^) followed by D_2_O:PBS (ρ = 1.101 g⋅cm^−3^). The median water content in these cells was 516±12 fg. We then measured cells sequentially in H_2_O:PBS (ρ = 1.005 g⋅cm^−3^) and D_2_O:PBS to obtain the dry mass distribution, yielding a median value of 203±5 fg. Finally, we measured the total mass distribution by the method of Grover et al. [14] and the median value was 727±15 fg. To ensure that the osmotic pressure experienced by the cells was equal in both fluids of each measurement, phosphate buffered saline (PBS) was added to the all the solutions in order to match their osmolarity.

The results presented above demonstrate that the method is self-consistent, as the water content of the cells plus the dry mass (sum of median values equals 719±13 fg) accounts for the total mass value (**Fig. 1c**). This suggests the median early stationary fixed *E. coli* cell is roughly 28% dry material by mass and 20% by volume, though these numbers may be different in living cells.

### Dry Density

#### Bacteria

**Figure 2:**
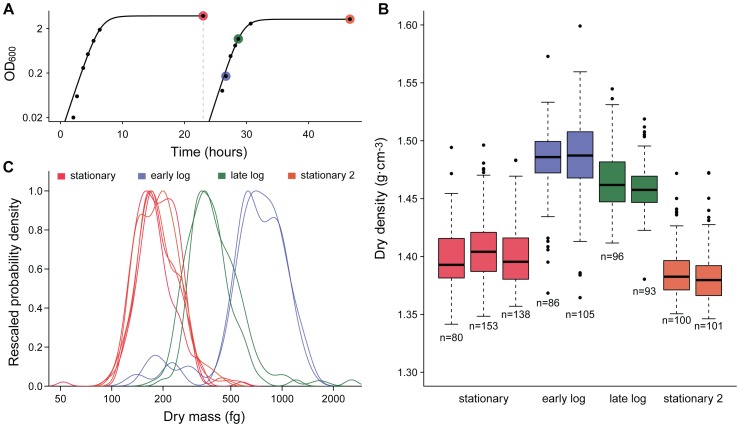
Dry density and dry mass of a bacterial culture. **a)** Growth curves of *E. coli* cultures. A culture was grown for 24 hours, diluted 1000-fold, and allowed to grow again for 24 hours. Samples from the cultures were fixed for analysis at the colored time-points. Solid line is the fit to logistic growth model. **b)** The dry density of the culture by sampling time point. Technical replicates of these fixed samples show that the changes in density are reproducible and not attributable to instrument error. **c)** Probability distributions of dry mass, rescaled so that the modal mass had a density of one. Lines of the same color are technical replicates, measured several days apart.

We investigated whether and how bacterial dry density and dry mass change with culture growth phases by growing *E. coli* cells and analyzing fixed samples of the culture at four time points - stationary, early exponential (after dilution into new culture), late exponential, and a second stationary point (**Fig. 2**). Each fixed sample was analyzed two to three times over several days to verify the results were consistent. We found that dry mass increased in early exponential phase, then rapidly decreased upon entry into stationary phase, which has been reported previously [5], [15]. Dry density exhibited a similar trend, initially increasing when stationary *E. coli* were diluted into fresh medium and entered exponential growth. As the culture progressed towards late exponential phase, the dry density decreased and by stationary phase had returned to the same value as the previous stationary culture. We also noted a subpopulation of cells in the early exponential sample that had masses and dry densities characteristic of stationary cells (left shoulder on blue distributions in **Fig. 2b**, see also **Fig. S2**).

Compared to the variation in total density reported previously for *E. coli* [16] and other microorganisms such as yeast [17], our single-cell dry density measurements were much more variable. To investigate the source of this variation, we looked at how errors in buoyant mass measurement would propagate to dry density estimates (**Fig. S3**) and simulated error distributions for our experimental conditions (see **Methods** and **Supporting Information**). For *E. coli* measurements, the simulated and observed distributions match quite well (**Fig. S4**), suggesting that the majority of the observed heterogeneity arises from buoyant mass measurement error. Thus, it is likely that the true dry density variation is lower than what we observe. We also note that the dry density may be affected by the fixatives needed for technical replicates.

#### Yeast

**Figure 3:**
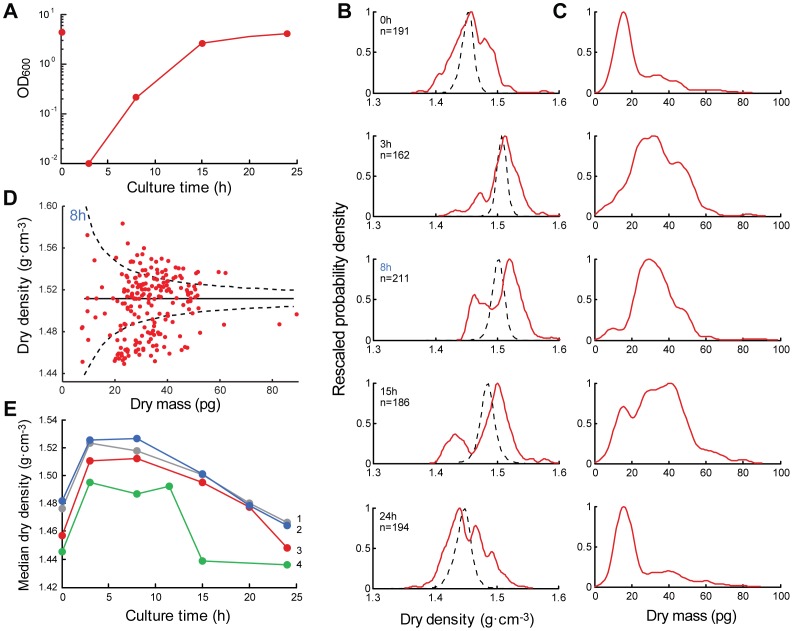
Dry density and dry mass of a yeast culture. **a)** Growth curve of a culture started from a 1000-fold dilution of a recently-saturated culture (time 0h). **b)** Distributions of dry densities for the time points indicated in a). Distributions expected due purely to measurement error (see text) are shown as black dashed lines. **c)** Dry mass distributions for the same time points. **d)** Single-cell data for time point 8h. Solid lines is median dry density and dashed lines are 99% bounds on the expected dry densities if all cells actually had the median dry density, given known measurement error (see **Methods**). **e)** Dry density distribution medians for several replicates: curves 1 and 2 are technical replicates and 2–4 are biological replicates; curve 3 is for data show in b).

We were interested in whether these patterns of changes were unique to bacteria, or if they might also be found in eukaryotic cells. As with *E. coli*, we grew a culture of yeast for a 24 h growth cycle, taking samples throughout the culture’s growth phases for fixation and quantification of their dry mass and dry density (**Fig. 3**). The measurements were repeated both with technical and biological replicates showing consistency amongst the measurements and trends (summarized in **Fig. 3e**).

Single-cell distributions are shown for dry density (**Fig. 3b**) and dry mass (**Fig. 3c**). The first time point during growth, 3h after the dilution, shows a concurrent increase in cell dry mass and dry density, when cells are growing at their fastest growth rate and actively dividing. The gradual decrease in dry density accompanies the slowing speed of culture growth as the culture approaches saturation. In contrast to the *E. coli* results, the computed error distributions are less variable than what we observe (**Fig. 3b**), showing true density heterogeneity and possibly distinct subpopulations. Additionally, we concurrently annotated cells as budded or unbudded by brightfield microscopy. In early stationary phase cultures, the dry density distribution of budded cells showed higher median values than of unbudded cells, but in exponential phase, the dry densities were not significantly different. (**Fig. S5**).

#### Mammalian cells

Finally, we measured the changes in dry density that occur when varying mammalian cells are subjected to similar changes in growth conditions. We chose four cell types – mouse embryonic fibroblasts, (MEFs), L1210 mouse lymphocytic leukemia cells, FL5.12 mouse prolymphocytic cells, and CD8 T cells from an OT-1 transgenic mouse – and manipulated the proliferative state of each. For MEFs, cells were grown either to 70% or 100% confluency. L1210 cells were treated with cycloheximide and measured before and 24 hours after treatment. FL5.12 cells were measured before and 20 hours after being placed in media lacking interleukin 3 (IL-3). Finally, naïve OT-1 CD8 T cells were activated with an ovalbumin peptide and measured before and 96 hours after activation. All measurements were performed without cell fixation.

**Figure 4:**
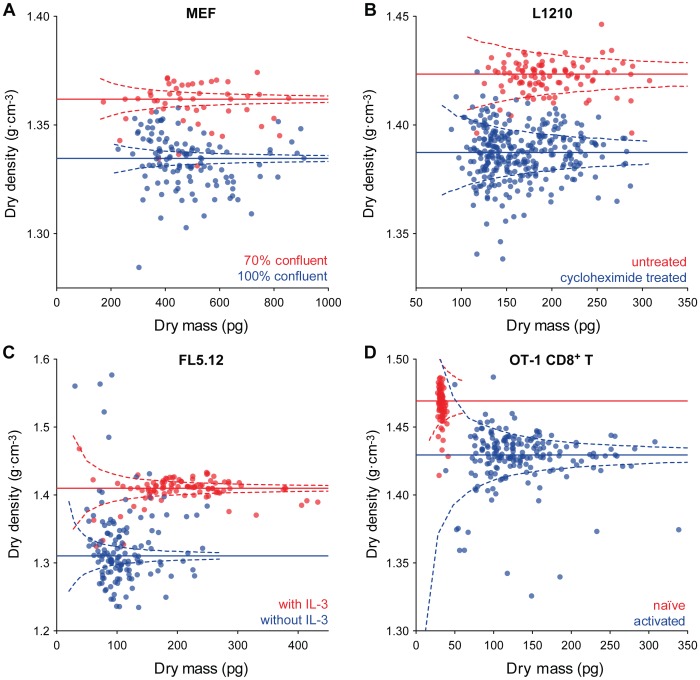
Dry density and mass of proliferating and non-proliferating mammalian cells. Solid lines are median dry densities and dashed lines are 99% bounds on the expected dry densities if all cells actually had the median dry density, given known measurement error. **a)** Confluent and proliferating (75% confluency) mouse embryonic fibroblasts. **b)** Cycloheximide-treated and proliferating L1210 cells. **c)** IL-3-depleted and proliferating FL5.12 cells. **d)** Naïve and activated OT-1 T cells.

With the exception of the activated OT-1 cells, proliferating cells appeared to have higher dry densities than their non-proliferating counterparts (**Fig. 4**). Moreover, both MEF cells grown to confluency (**Fig. 4a**) and L1210 cells treated with cycloheximide (**Fig. 4b**) did not show a substantial decrease in dry mass relative to steady state populations. FL5.12 cells, however, decreased both in dry density and dry mass when starved of IL-3 (**Fig. 4c**). Interestingly, primary OT-1 T CD8 cells, which are quiescent and non-proliferating (naïve), had higher dry density prior to activation than following activation (**Fig. 4d**). Of these four mammalian cell lines, only for the naïve OT-1 T cells is the variation nearly completely accounted for by measurement error, suggesting non-negligible biological variation in the other populations. Additionally, for all the cells except the OT-1 cells, the observed variation in dry density increased upon interfering with proliferation.

#### Red blood cells

**Figure 5:**
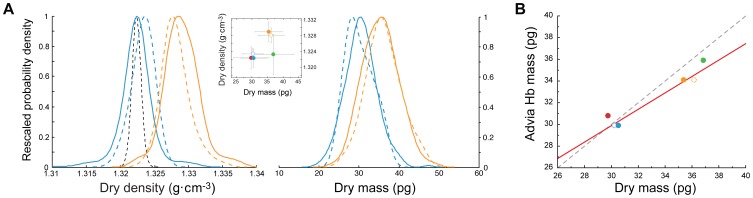
Dry density and mass of red blood cells. **a)** Single-cell dry density and dry mass distributions of two different human erythrocyte samples. Solid and dashed lines of same color indicate technical replicates. Dashed black line is a 99% bound on the expected dry density of a representative sample if all cells had the median dry density, given known measurement error. **inset** Population mean values from four patient samples. Error bars are standard deviation of the population. **b)** Comparison to hemoglobin mass per cell determined with an Advia instrument. Dashed line indicates y = x and solid line is a total least squares fit.

Human erythrocytes are a unique sample for our method because they are deformable enough that we can flow them through sensitive 3×5 µm channel devices designed for bacteria. No cell lysis was observed, consistent with reports of unimpeded flow of red blood cells through 3 µm diameter pores [18]. As a result, there is essentially no error caused by variability in cell transit flow paths, and because they are 40 to 160 times larger than bacteria, the signal-to-noise ratio is higher than for any other sample. From four different human samples, we find that erythrocytes have extremely narrow dry density distributions (median sample standard deviation of 0.0024 g⋅cm^–3^, maximum 0.0051 g⋅cm^–3^) and the measurements are highly reproducible (**Fig.** **5a**). The narrowness of the dry density distributions allow us to distinguish differences in dry density amongst different populations that may or may not have distinct dry mass distributions. We also compared the dry mass of the red blood cells, with the mean hemoglobin content quantified by the FDA-approved Siemens ADVIA instrument (**Fig. 5b**).

## Discussion

We have introduced a non-optical technique for quantifying the dry mass, water content and dry density of either living or fixed cells that does not require any assumptions about the cell’s composition. However, it does rely on two key assumptions. First, to avoid osmotic perturbations that might damage or lyse a cell, the measurements are not made in pure H_2_O and D_2_O, but in isotonic solutions. Therefore the assumption that the intracellular water volume is exactly neutrally buoyant in the immersion fluid is an approximation, as there will be a difference between the densities of the intracellular water (or deuterium oxide) and of the fluids in which the cell is immersed. However, assuming the cell volume is 80% water, this error will be small (<0.04 g⋅cm^–3^ - see **Supporting Information** and **Fig. S6**). Knowing the exact fraction of water content would allow us to correct for this effect. Second, we assume that complete exchange of intracellular water occurs. This is justified, as we observe at most very weak (and typically statistically insignificant) correlations between dry density and the time a cell spends immersed in D_2_O (**Supporting Information** and **Figs. S7** and **S8**). Indeed, previous measurements of water permeation and diffusion across the membrane have demonstrated the almost instantaneous nature of the event [1].

Our dry mass results are consistent with previous reported measurements of the described single-cell or bulk methods. For instance, two TEM-based studies found median *E. coli* dry masses of 489 fg and 710 fg for exponentially growing cells and 179 fg and 180 fg for stationary ones [5], [7]. We report median masses of 725 fg and 179 fg, respectively, pooling technical replicates shown in **Figure 2**. Budding yeast dry mass content per cell is not widely reported and growth conditions vary can vary widely, but our results are in line with the values reported by Mitchison [6]. Further, the results for the human erythrocytes agree well with the hemoglobin content concurrently quantified by the FDA-approved ADVIA instrument (**Fig. 5b**), or by QPM [19]. It should be noted that, even though the ADVIA measures only hemoglobin content, this protein has been shown to account for more than 95% of the cell’s dry mass [20], [21]. By comparison to our results, the mean hemoglobin content determined by the ADVIA accounts for 97.7±1.3% of the total dry mass content.

A unique aspect of our measurement is the concurrent determination of dry density. The few mentions of this parameter in the literature are seemingly limited to measurements of wet and dry spores [12], [13], [22] or an application in an H_2_O/D_2_O density gradient [23]. However, none of these reports connect dry density to chemical composition of the dry content. Our results suggest that dry density is a direct manifestation of the changes in chemical composition of a cell’s biomass and we demonstrate that the parameter can be measured for single cells. While related to total cell density, dry density is independent of the intracellular water content and should not be perturbed by uptake or expulsion of water. In contrast, since the majority of a cell’s volume is composed of water, total density is likely to be much more indicative of changes in cell water content.

For *E. coli* [2], [15], [24] and yeast [3], [4], the RNA/protein ratio has been extensively correlated with growth rate: faster growing cells have an increased proportion of RNA relative to protein. We observe these growth-rate-dependent changes in chemical composition directly as changes in dry density, since the average density of proteins is lower than of RNA (**Table 2**). Faster growing cells – in early exponential phase – have a higher dry density, consistent with higher RNA/protein ratio, and as growth rate diminishes, so does dry density. Furthermore, the annotation of budded and unbudded yeast populations also reveals that at early saturation time points, budded cells tend to have higher dry densities than unbudded cells. However, as the culture enters exponential phase, the unbudded cells no longer possess a distinctive dry density profile. We speculate that the dry density variation results from heterogeneous proliferative states within a well-mixed culture - cells with higher dry densities are those that are currently or were more recently proliferating.

Our results with mammalian cells demonstrate that dry density changes when growth is perturbed. Both MEF and FL5.12 cells decrease their dry density as they transition into unfavorable growth conditions characterized by culture crowding or nutrient deprivation from IL-3 depletion. The decrease in MEF dry density can be compared to the results reported by Short et al. [25] for fibroblast derived M1 cells, in which the relative amounts of RNA and protein content decrease and lipid content increases for overcrowded non-proliferating cells when compared to proliferating ones. Changes in dry density are not necessarily correlated with dry mass. L1210 cells treated with a lethal dose of cycloheximide – which blocks protein synthesis – show a decrease in dry density even though the overall dry mass distribution does not show a substantial decrease. This suggests that during this period the cell has no notable net mass exchange with its environment, but the inner constituents of the cell are undergoing substantial biochemical alterations. As fewer proteins are synthesized during this time, degradation is likely the primary force lowering the relative protein content [26]. This increases the relative contribution of lower density components, such as lipids, thereby decreasing the overall dry density. The alteration of dry density in these situations suggests that this parameter can be indicative of cell proliferative state. If proliferating cells amongst a steady state population have different dry densities, dry density could be complementary to proliferation detection assays such as Ki-67 labeling.

We also wished to see if changing mammalian cell growth rate by activating growth from a natural quiescent state would result in a change in dry density. In their naïve state, CD8 T cells are quiescent and only begin proliferating following antigen stimulation. Comparison of naïve and activated OT-1 CD8 T cells show that T cell activation is accompanied by dramatic changes in both dry density and dry mass, suggesting, as in previous results, that as cells alter their proliferative state, changes in their chemical composition occur. It is notable that naïve CD8 T cells freshly isolated from mice show high dry density but very low dry mass. Stimulation of these cells into proliferation is associated with an expected increase in dry mass as well as a change in dry density to similar values of cultured L1210 cells. The increase in dry mass is consistent with the growth of the cells as they undergo proliferation. However, the high dry density of naïve CD8 T cells was unexpected, in light of the observation that the dry density decreases when mammalian cells undergo growth arrest due to stress or depletion of nutrition. Naïve CD8 T cells are compact with little cytoplasm –consistent with their low dry mass value – and their high dry density may reflect the lack of cytoplasm and organelles, which are rich in lipids. As a result, the proportion of nucleic acids and proteins is higher for naïve T cells than actively dividing T cells.

Finally, we demonstrate that the concept of rapid exchange of intracellular water can be used to quantify a cell’s water content. Although we can currently measure only an average or modal intracellular water fraction, the ability to weigh single cells in three different fluids will allow all of the described quantities to be determined simultaneously.

## Materials and Methods

### Ethics Statement

Human erythrocytes samples were collected from subjects under a discarded specimen protocol approved by the Partner Human Research Committee, Partners Human Research Office, 116 Huntington Avenue, Suite 1002 Boston, MA 02116. Blood specimens were de-identified, and the ethics committee waived the requirement for informed consent after determining that the risk to subjects was minimal. The research protocol and research progress were reviewed and approved upon initial submission and every two years thereafter by the ethics committee.

All experiments with mice were performed in accordance with the institutional guidelines approved by the Massachusetts Institute of Technology Committee on Animal Care (CAC), which specifically approved the animal part of this study.

### SMR Operation

Three types of SMRs were used to perform the measurements. For bacteria and red blood cells, the procedure is identical to that of Grover et al. [14] but smaller 3×3×100 µm and 3×5×120 µm (channel height × width × length, for bacteria and red blood cells respectively) cantilevers were used. In some experiments OptiPrep (iodixanol) was used instead of Percoll. Budding yeast cells were measured as previously described for single-cell density by Weng et al. [17], with an 8×8 µm, 210 µm-long three-channel device. Finally, the larger mammalian cells were measured with a 15×20 µm, 450 µm-long channel SMR with the Grover et al. method, however the device was being operated in the second vibrational mode [27].

To measure dry mass and dry density, the cells are weighed twice, first in a water-based solution (1X PBS in H_2_O), secondly in a deuterium oxide-based solution (1X PBS in 9∶1 D_2_O:H_2_O) (**Fig. S1**). The actuation of the cantilever in the second vibrational mode increases sensitivity and decreases error by eliminating the flow path dependency of the buoyant mass measurement. The three-channel devices also eliminate this error, as described elsewhere [27]. However, technical constraints prevent us from using either of these methods with the smaller bacterial-sized devices.

After the first weighing, the cell is immersed in the second fluid and the two measurements can be done several seconds apart. The fluidic exchange occurs on a faster time-scale. While it is difficult to make the two measurements faster in less than several seconds using regular cantilevers, with the three-channel devices the fluid environment can be switched from H_2_O to D_2_O very rapidly (250 ms). During this time, depending on the device, the cell is either outside of the sensor (all samples but yeast), or in trapped inside it (yeast). However, even in the case of yeast, we cannot directly observe the time dynamics of intracellular water exchange because it is obscured by the large transient signal resulting from changing the fluid in the cantilever.

To measure water content in single *E. coli*, cells were initially immersed in a solution of roughly 18% OptiPrep (w/v), 0.9X PBS in H_2_O. Because it is essential that the fluid densities match precisely, this solution density was manually adjusted with a few drops of water or 60% OptiPrep to match the density of 1X PBS in 9∶1 D_2_O:H_2_O. Cell buoyant masses were then sequentially measured in the OptiPrep:PBS:H_2_O solution, followed by the PBS:D_2_O solution.

SMR buoyant mass measurements were calibrated using polystyrene particles of varying sizes (depending on SMR type) from Duke Scientific and from Bangs Labs. Fluid density measurements were calibrated with NaCl standard solutions. All measurements were done at 22–23°C.

### Cell Culture and Fixation

#### Escherichia coli

Cells (ATCC 23725) were grown on Luria Broth (LB) agar plates from frozen stock, and single colonies were transferred into 35 mL liquid cultures (LB) and grown for 24 hours at 37°C with vigorous shaking. Two cultures were grown to verify similar growth behavior by optical density at 600nm. After 24 hours, several milliliters from one culture were fixed in 2% paraformaldehyde and 2.5% glutaraldehyde for 1 hour at 4 C. Some of the same culture was also used to inoculate a 35 mL culture at a 1000-fold dilution. Cells were then taken at OD_600_ 0.17 and 1.17 and fixed (220 minutes and 340 minutes, respectively), and then a final sample was fixed at 24 hours (OD_600_ ∼3.3).

#### Saccharomyces cerevisiae

Haploid cells (702 W303, strain A2587 [28]) were grown in YEPD medium at 30°C, well-shaken. For suspended culture growth experiments cells were started from a plated culture and grown for 24 h. At that point an aliquot was sampled and a new culture was started with a 1000-fold dilution. Subsequent aliquots were sampled at the times described in the text. Each sample was spun down, suspended in PBS, sonicated and fixed in 4% paraformaldehyde overnight.

#### Red blood cells

Four human erythrocytes samples were collected in EDTA from subjects under a research protocol approved by the Partners Healthcare Institutional Review Board. Samples were diluted in PBS prior to each dry measurement. “Advia” hemoglobin mass measurements were performed on a Siemens Advia 2120 instrument.

#### L1210

L1210 murine lymphoblasts (ATCC CCL-219) cells were grown at 37°C in L-15 media supplemented with 0.4% (w/v) glucose, 10% (v/v) fetal bovine serum (FBS), 100 IU penicillin and 100 µg/mL streptomycin. For cycloheximide treatment, 5 µL of a 10 mg/mL cycloheximide in DMSO stock solution were added to a 5 mL culture (7.5 × 10^5^ cells/mL) of L1210 cells. Treated cells were maintained in an incubator at 37°C for 24 hours prior to measurement. Before loading the sample into the SMR, cells were washed twice in PBS by spinning down for 5 minutes each time at 100 RCF. The concentration of the cell sample was adjusted to 5 × 10^5^/mL.

#### FL5.12

Cells were grown at 37°C in RPMI media supplemented with 10% (v/v) FBS, 100 IU penicillin, 100 µg/mL streptomycin and 0.02 µg/mL IL-3. For FL-5.12 starvation, a confluent culture of FL5.12 cells (10^6^/mL) was washed three times before culturing for 20 h in RPMI media lacking IL-3. Before measurement in the SMR, cells were washed twice with PBS as with the L1210 cells. FL5.12 are a murine pro-B-cell lymphoid cell line and were a gift from gift from Matt Vander Heiden (MIT) and cultured as previously described [29].

#### Mouse endothelial fibroblasts

Cells were grown at 37°C in DMEM media supplemented with 10% (v/v) FBS, 100 IU penicillin and 100 µg/mL streptomycin. Cells were trypsinized and measured at 70% confluency (10^6^ cells on a 25 cm^2^ flask) or overconfluency (2 × 10^6^/mL). Cells were washed twice with PBS before loading into the SMR. MEFs were a gift from Denis Wirtz (Johns Hopkins University) [30].

#### OT1 CD8 T cells

Lymph nodes were harvested from OT1-rag1^−/−^ mice, ground and filtered using a 70 µm nylon cell strainer. To activate T cells, OT-1 cells were stimulated with 2 µg/mL OVA_257−264_ peptide (SIINFEKL) at 37°C for 24 hours in RPMI media supplemented with 10% (v/v) FBS, 100 IU penicillin, 100 µg/mL streptomycin, 2 mM L-glutamine, 1 mM sodium pyruvate, 55 µM 2-mercaptoethanol and 100 µM nonessential amino acids, followed with culture for another 4 days in the presence of 50 IU IL-2. Cells were washed twice with PBS before measurements. Measurements of naïve CD8 T cells were carried out immediately after harvesting from mice. All experiments with mice were performed in accordance with the institutional guidelines.

### Statistical Analysis

To estimate uncertainty in dry density and dry mass measurements, we first estimate the uncertainty in buoyant mass measurements, and then simulate how this measurement error propagates through the density and mass calculations. Buoyant mass uncertainty is estimated from the discrepancy between two sequential measurements of a cell in the same fluid, as the two measurements are expected to be identical. Two sequential measurements are made from approximately 100 cells, and for each cell, the difference between the two measurements is calculated. As the difference in each pair of measurements is the difference of two presumed independent errors, rescaling the distribution of differences by 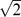 yields approximately the distribution of errors that might occur on a single buoyant mass measurement.

For dry mass, the standard error in an estimate is approximately 15 times greater than the error in a single buoyant mass measurement (see **File S1** for calculations). However, dry density is a non-linear function of the two buoyant mass measurements and so we simulate the effect of buoyant mass errors on a population with no dry density variability. We begin by assuming all particles have a density equal to the median observed dry density and a mass distribution equal to our observed dry mass distribution. We sample 10000+ hypothetical particle masses from our observed dry mass distribution and calculate buoyant masses for those particles in the two fluids used for the experiment. We then sample errors from our measured error distribution, add this random noise to each buoyant mass measurement, and calculate the dry density for each ‘noisy’ pair of buoyant mass measurements. Although no dry density heterogeneity went into this calculation, the resulting dry density measurements have a non-zero variation due to buoyant mass errors, and we qualitatively compare these distributions to our observed dry density distributions.

## Supporting Information

Figure S1 Using the SMR to measure the buoyant mass of a cell in H_2_O and D_2_O. The measurement starts with the cantilever filled with H_2_O (blue, box 1). The density of the red fluid is determined from the baseline resonance frequency of the cantilever. When a cell passes through the cantilever (box 2), the buoyant mass of the cell in water is measured as a transient change in resonant frequency. The direction of fluid flow is then reversed, and the resonance frequency of the cantilever changes as the cantilever fills with D_2_O, a fluid of greater density (red, box 3). The buoyant mass of the cell in D_2_O is measured as the cell transits the cantilever a second time (box 4). From these four measurements of fluid density and cell buoyant mass, the absolute mass, volume, and density of the cell’s dry content are calculated. (Adapted from Grover *et al*. [14]).
(TIF)

Figure S2 Dry mass *versus* dry density of single *E. coli* cells. Same data as shown in **Figure 2**, but plotted to show single cells rather than just marginal distributions.
(TIF)

Figure S3 a) Contour map of density as a function of two buoyant mass measurements. b) In polar coordinates, the angle can be shown to map directly to density. c) Contour map showing cell mass as a function of two buoyant masses. This function is linear, with a gradient oriented to the lower right (higher buoyant mass in H_2_O, lower buoyant mass in D_2_O).
(TIF)

Figure S4 Comparison of measured data (solid lines) to simulations of buoyant mass measurement errors propagating through the density calculation for *E. coli* samples. Dashed lines show expected dry density distributions assuming all cells have the same density and that density is the median observed dry density (vertical line).
(TIF)

Figure S5 Dry density distributions for budded and unbudded yeast cells, by timepoint. P-values are for two-sided Mann-Whitney U tests.
(TIF)

Figure S6 Contour plots of dry density estimates when the buoyant mass measurements aren’t made in pure H_2_O or pure D_2_O. Intracellular water fractions are in fraction of total volume. Dashed line shows equal departure (in density) from pure fluids. Pure H_2_O and 9∶1 (v/v) D_2_O:H_2_O densities are the red dot in the lower left corner of each figure, at which point the dry density is calculated correctly. As salts (or other impermeable components) are added to the fluid, it becomes more dense and the intracellular water is no longer neutrally buoyant. This introduces systematic error into the dry density measurement, which depends on how much of the cell is water. The measurements we’ve made using 1× PBS in both fluids are shown as black dots.
(TIF)

Figure S7 Time between measurements (exposure time) *versus* calculated dry density for single cells in each of nine analyses of *E. coli* samples (2–3 technical replicates for each of 4 samples). Assuming the cell was nearly immediately immersed in D_2_O after the first measurement, this should be a good approximation of time spent in D_2_O. Line shows ordinary least squares fits, which agreed well with robust fits (Huber weights). Correlations are all statistically insignificant at α = 0.05 (α = 0.006 for each test, using Bonferroni correction). P-values are given for slope being non-zero using one-sided t-test.
(TIF)

Figure S8 Time between measurements (exposure time) *versus* calculated dry density for single *S. cerevisiae* cells in four experiments. Line shows ordinary least squares fits, which never account for more than 5% of the total variance. Because these experiments were done three-channel devices, much more precise control over exposure time could be achieved, and this parameter was deliberately varied, yielding the discrete times seen above. Only one experiment showed a statistically significant correlation (α = 0.05/4 = 0.0125 using Bonferroni correction). P-values are given for slope being non-zero using one-sided t-test.
(TIF)

File S1 Supplemental discussion: error sources, evidence for complete fluid exchange, description of water-content measurement method and remarks on the necessity of single-cell measurements.
(PDF)
