# Correction: Intracellular Water Exchange for Measuring the Dry Mass, Water Mass and Changes in Chemical Composition of Living Cells

**DOI:** 10.1371/annotation/c3a3219b-935b-42ed-b3a7-bbbc36dc1dfe

**Published:** 2013-09-17

**Authors:** Francisco Feijó Delgado, Nathan Cermak, Vivian C. Hecht, Sungmin Son, Yingzhong Li, Scott M. Knudsen, Selim Olcum, John M. Higgins, Jianzhu Chen, William H. Grover, Scott R. Manalis

The following information was missing from the funding section: NSF award #1129359. 

