# Supplementary figures and images for: Intracellular Water Exchange for Measuring the Dry Mass, Water Mass and Changes in Chemical Composition of Living Cells
Source: PLoS One. 2013 Jul 2;8(7):e67590. doi: 10.1371/journal.pone.0067590 (PMC3699654; doi:10.1371/journal.pone.0067590)

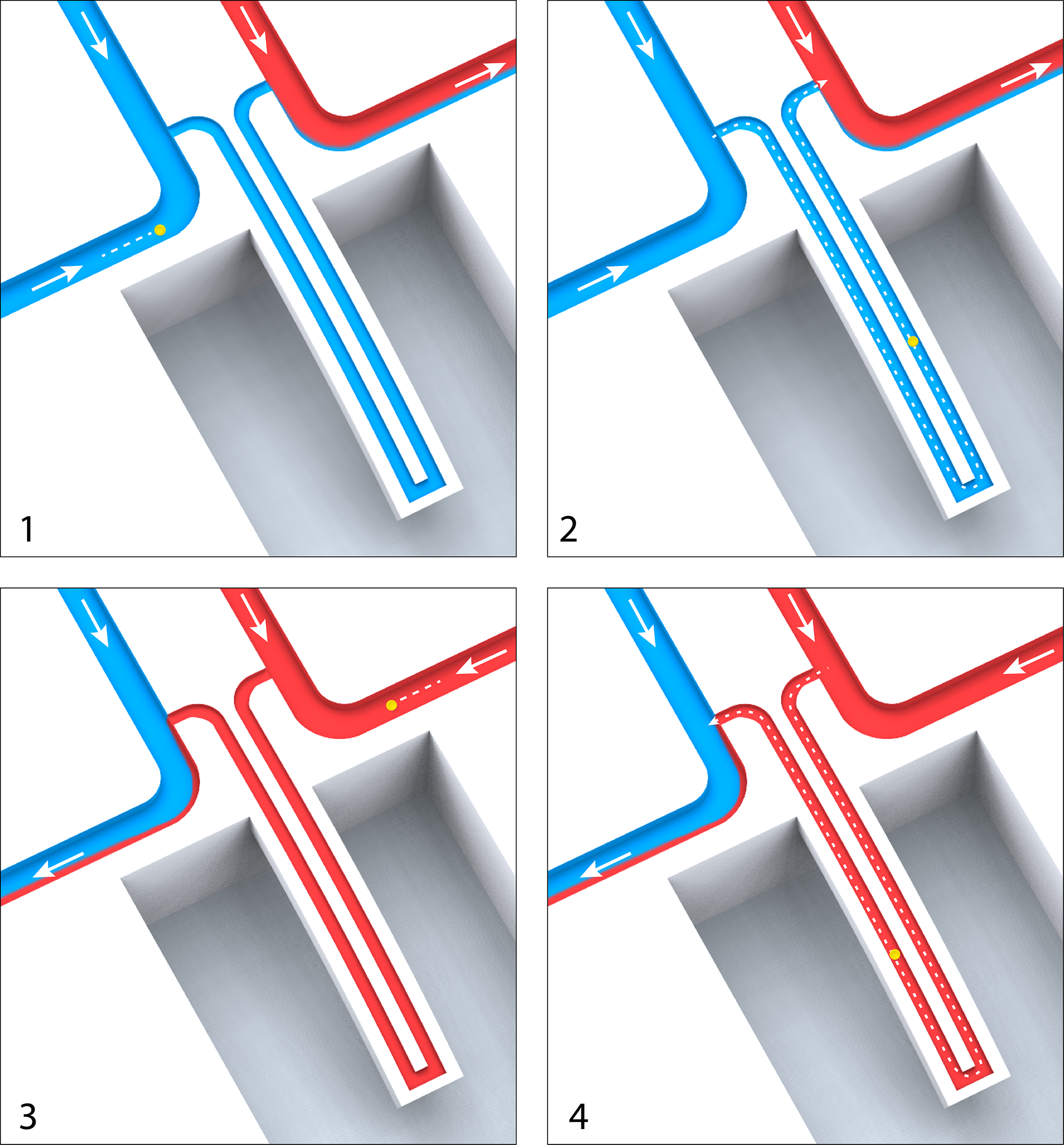

Supplement: Figure S1 — Using the SMR to measure the buoyant mass of a cell in H2O and D2O. The measurement starts with the cantilever filled with H2O (blue, box 1). The density of the red fluid is determined from the baseline resonance frequency of the cantilever. When a cell passes through the cantilever (box 2), the buoyant mass of the cell in water is measured as a transient change in resonant frequency. The direction of fluid flow is then reversed, and the resonance frequency of the cantilever changes as the cantilever fills with D2O, a fluid of greater density (red, box 3). The buoyant mass of the cell in D2O is measured as the cell transits the cantilever a second time (box 4). From these four measurements of fluid density and cell buoyant mass, the absolute mass, volume, and density of the cell’s dry content are calculated. (Adapted from Grover et al. [14]). (TIF) [file pone.0067590.s001.tif]

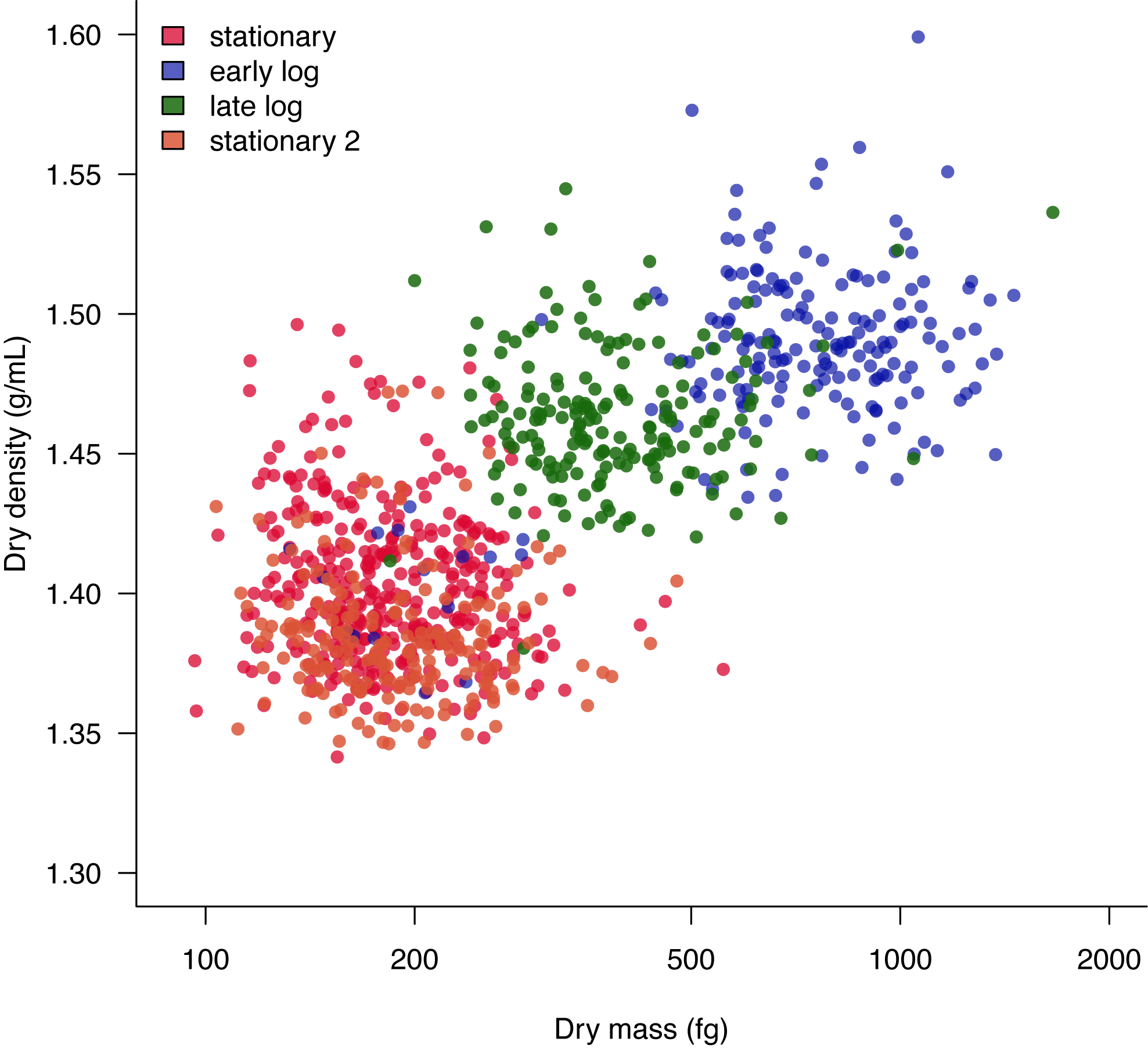

Supplement: Figure S2 — Dry mass versus dry density of single E. coli cells. Same data as shown in Figure 2 , but plotted to show single cells rather than just marginal distributions. (TIF) [file pone.0067590.s002.tif]

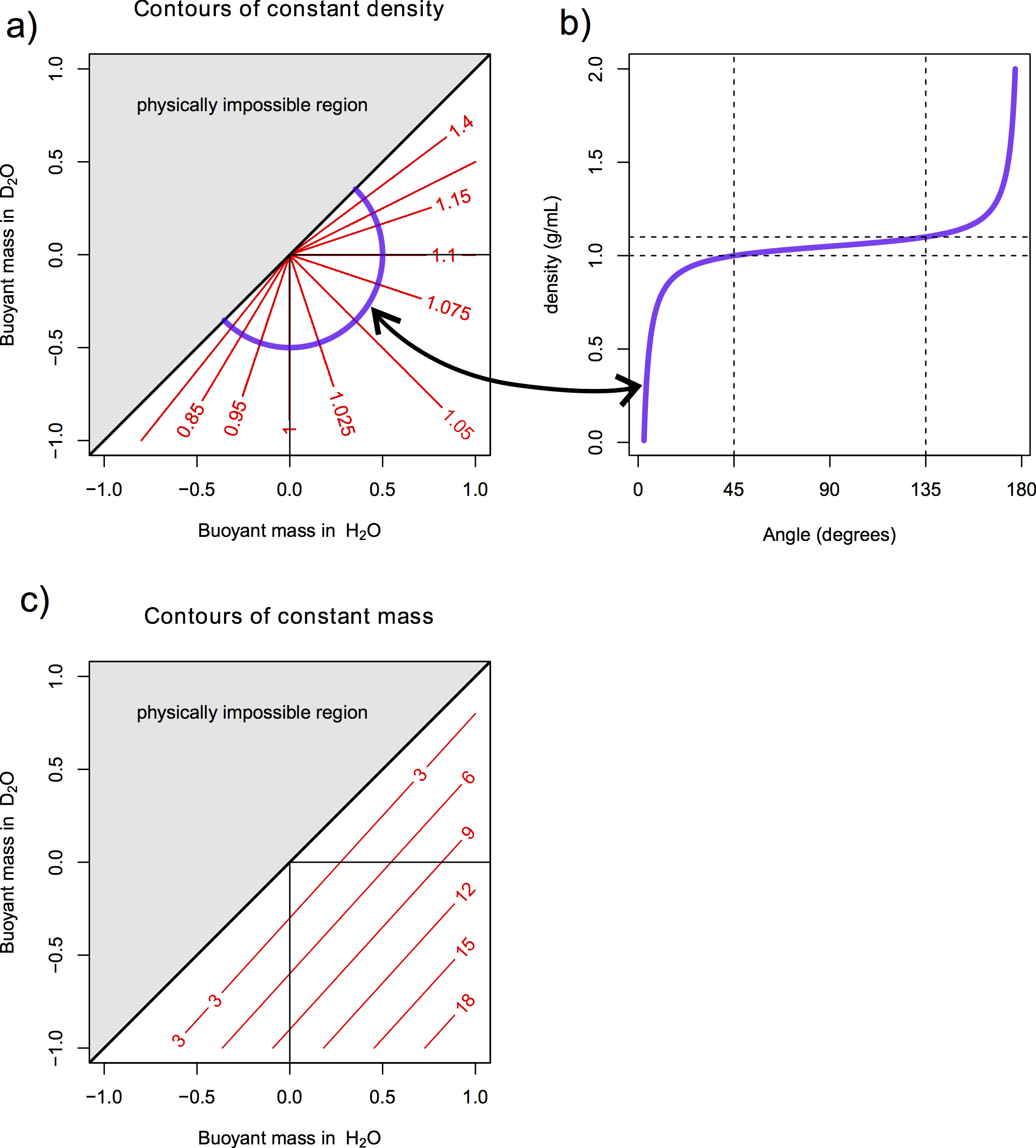

Supplement: Figure S3 — a) Contour map of density as a function of two buoyant mass measurements. b) In polar coordinates, the angle can be shown to map directly to density. c) Contour map showing cell mass as a function of two buoyant masses. This function is linear, with a gradient oriented to the lower right (higher buoyant mass in H2O, lower buoyant mass in D2O). (TIF) [file pone.0067590.s003.tif]

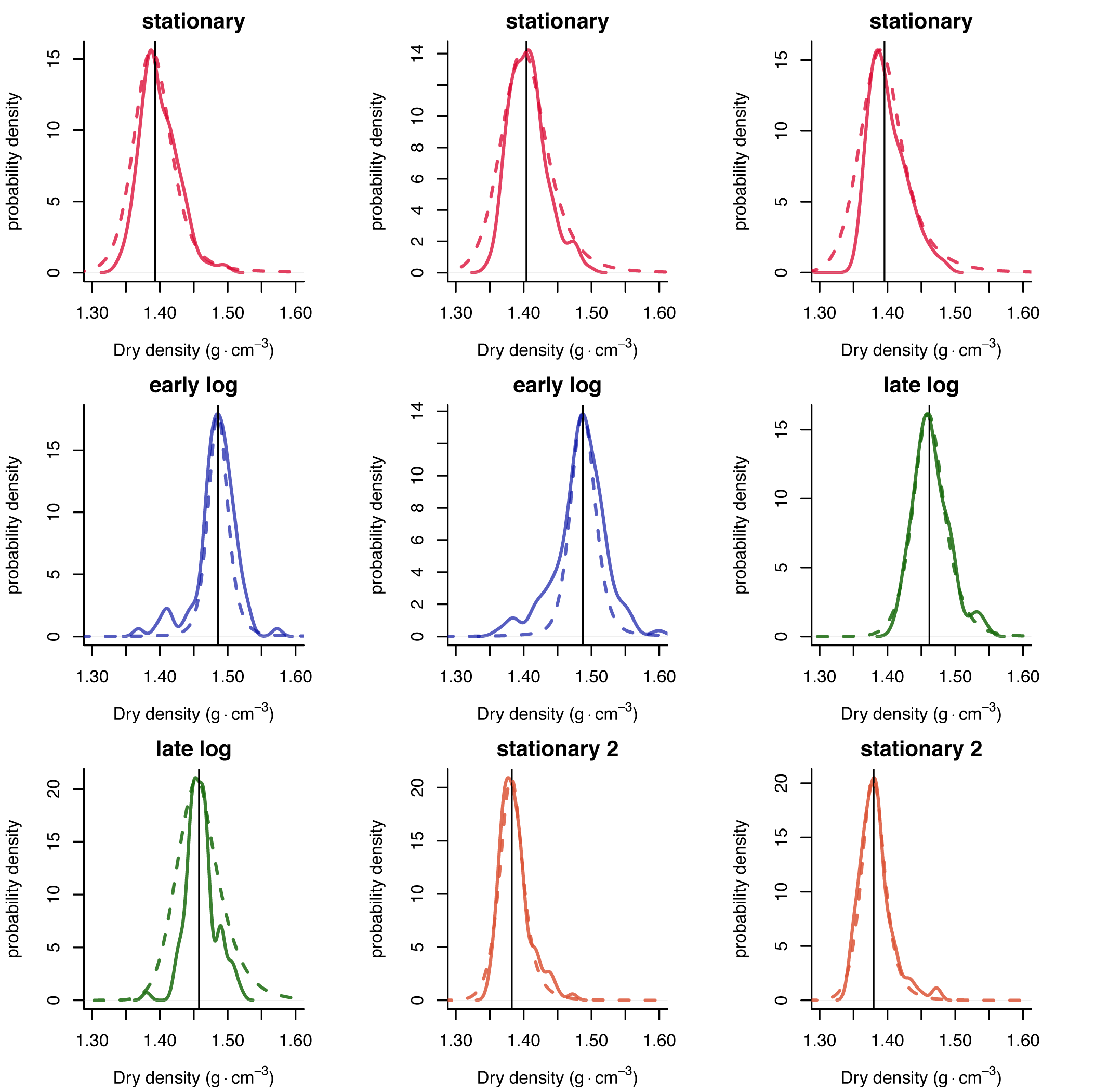

Supplement: Figure S4 — Comparison of measured data (solid lines) to simulations of buoyant mass measurement errors propagating through the density calculation for E. coli samples. Dashed lines show expected dry density distributions assuming all cells have the same density and that density is the median observed dry density (vertical line). (TIF) [file pone.0067590.s004.tif]

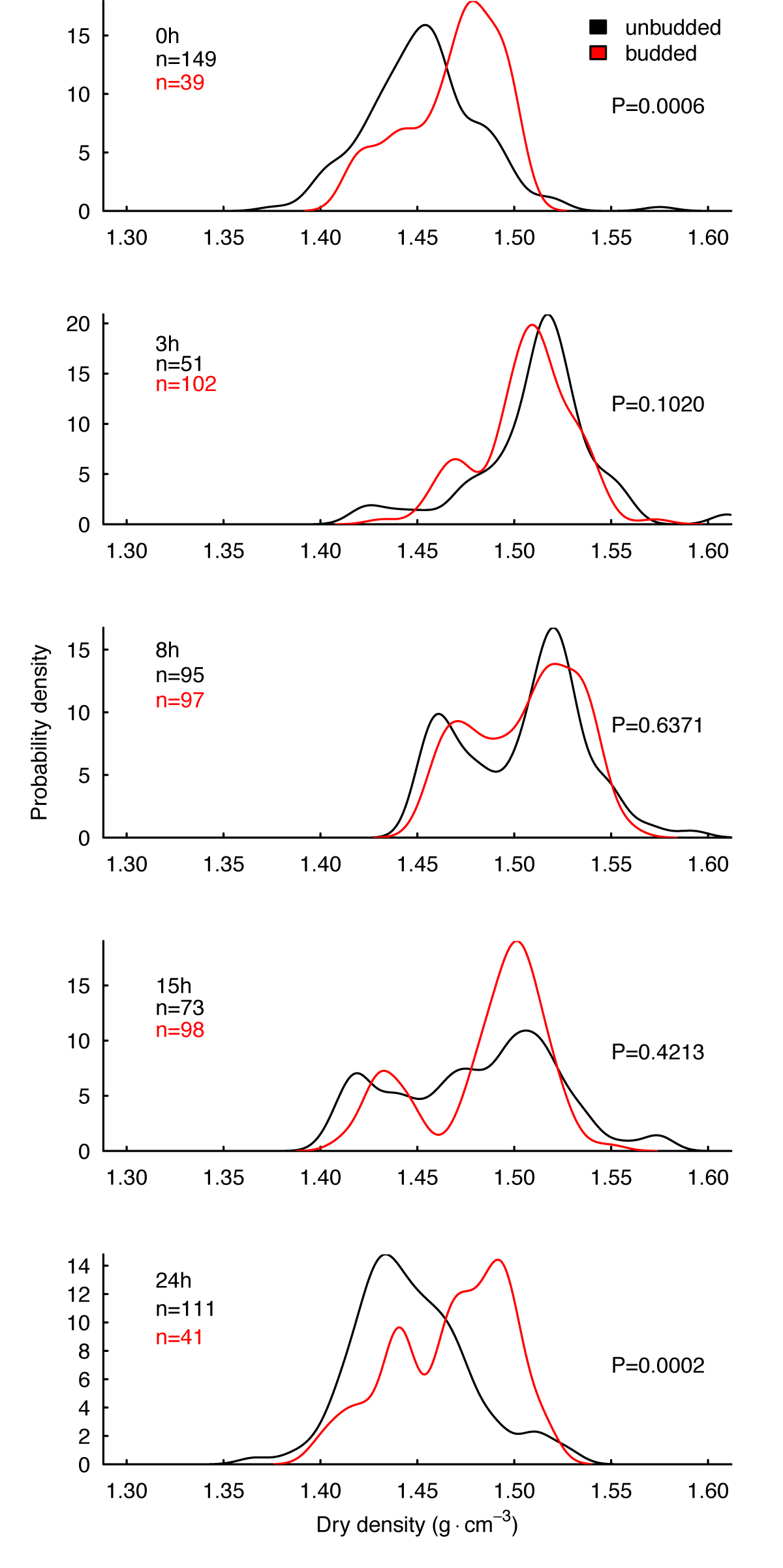

Supplement: Figure S5 — Dry density distributions for budded and unbudded yeast cells, by timepoint. P-values are for two-sided Mann-Whitney U tests. (TIF) [file pone.0067590.s005.tif]

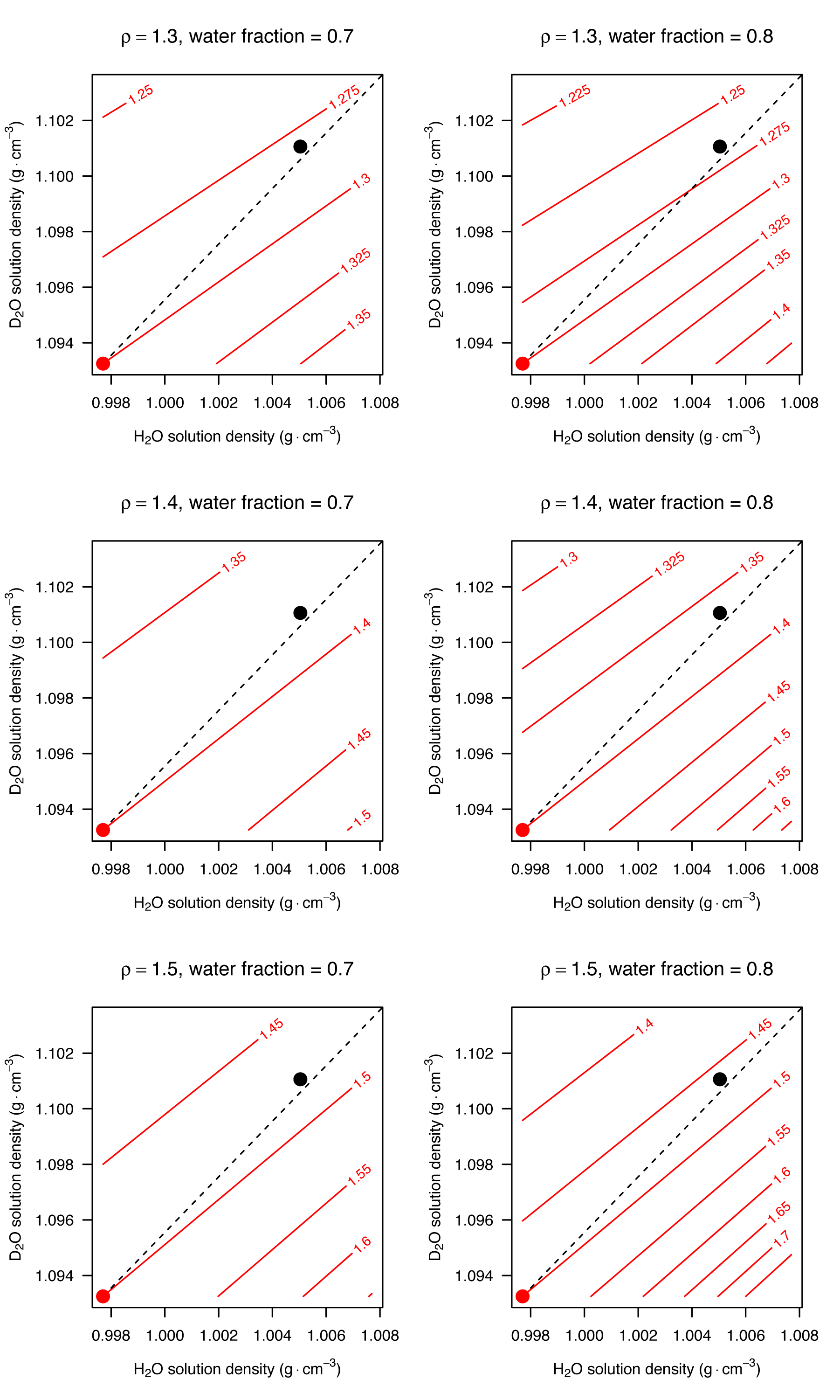

Supplement: Figure S6 — Contour plots of dry density estimates when the buoyant mass measurements aren’t made in pure H2O or pure D2O. Intracellular water fractions are in fraction of total volume. Dashed line shows equal departure (in density) from pure fluids. Pure H2O and 9∶1 (v/v) D2O:H2O densities are the red dot in the lower left corner of each figure, at which point the dry density is calculated correctly. As salts (or other impermeable components) are added to the fluid, it becomes more dense and the intracellular water is no longer neutrally buoyant. This introduces systematic error into the dry density measurement, which depends on how much of the cell is water. The measurements we’ve made using 1× PBS in both fluids are shown as black dots. (TIF) [file pone.0067590.s006.tif]

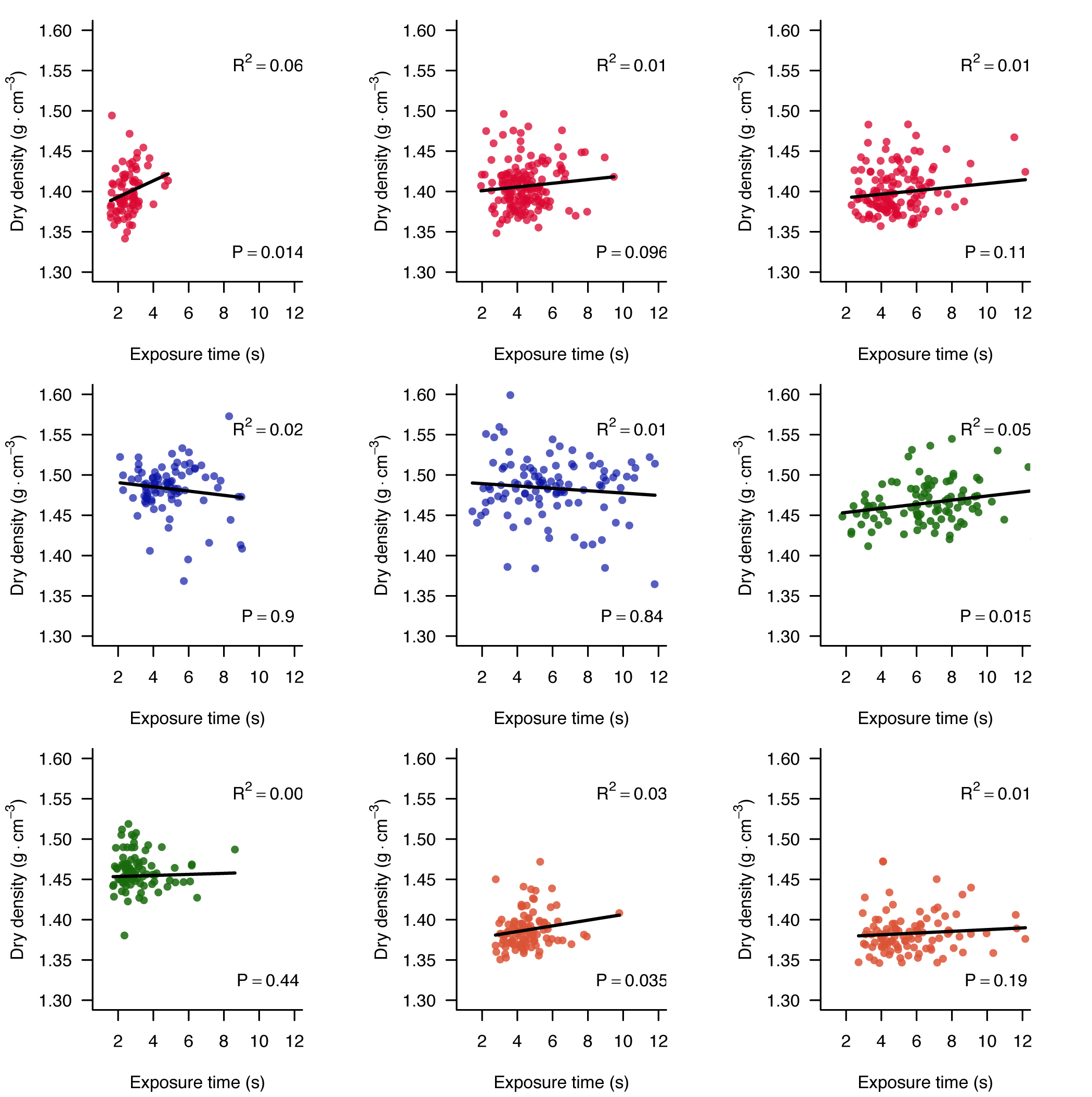

Supplement: Figure S7 — Time between measurements (exposure time) versus calculated dry density for single cells in each of nine analyses of E. coli samples (2–3 technical replicates for each of 4 samples). Assuming the cell was nearly immediately immersed in D2O after the first measurement, this should be a good approximation of time spent in D2O. Line shows ordinary least squares fits, which agreed well with robust fits (Huber weights). Correlations are all statistically insignificant at α = 0.05 (α = 0.006 for each test, using Bonferroni correction). P-values are given for slope being non-zero using one-sided t-test. (TIF) [file pone.0067590.s007.tif]

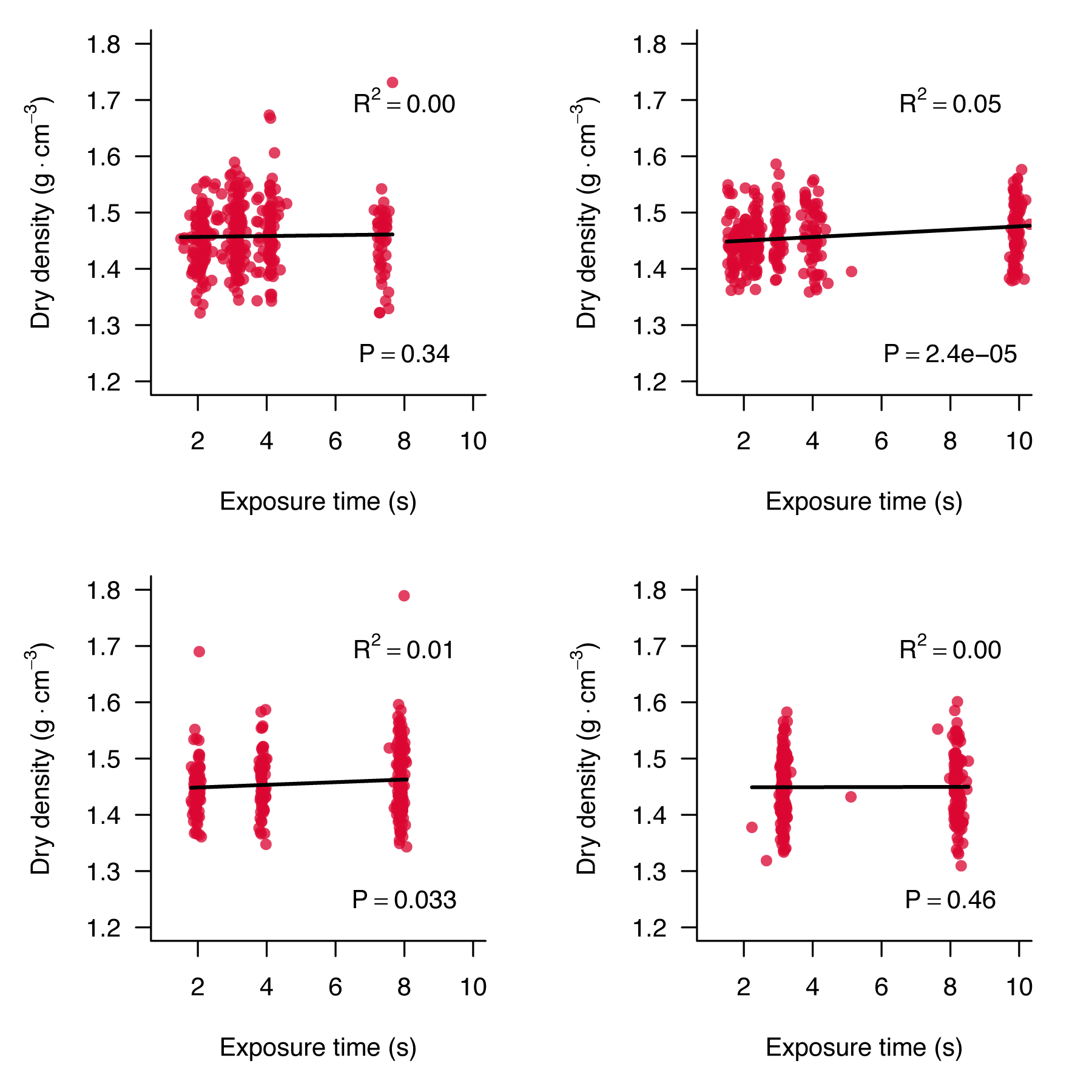

Supplement: Figure S8 — Time between measurements (exposure time) versus calculated dry density for single S. cerevisiae cells in four experiments. Line shows ordinary least squares fits, which never account for more than 5% of the total variance. Because these experiments were done three-channel devices, much more precise control over exposure time could be achieved, and this parameter was deliberately varied, yielding the discrete times seen above. Only one experiment showed a statistically significant correlation (α = 0.05/4 = 0.0125 using Bonferroni correction). P-values are given for slope being non-zero using one-sided t-test. (TIF) [file pone.0067590.s008.tif]
