# Supplementary material for: Intracellular Water Exchange for Measuring the Dry Mass, Water Mass and Changes in Chemical Composition of Living Cells
Source: PLoS One. 2013 Jul 2;8(7):e67590. doi: 10.1371/journal.pone.0067590 (PMC3699654; doi:10.1371/journal.pone.0067590)
Supplement: File S1 — Supplemental discussion: error sources, evidence for complete fluid exchange, description of water-content measurement method and remarks on the necessity of single-cell measurements. (PDF) [file pone.0067590.s009.pdf]

Supporting Information

To obtain mass, volume, and density, we require buoyant mass measurements in two fluids of different densities:

$$\begin{bmatrix} m_{b_1} \\ m_{b_2} \end{bmatrix} = \begin{bmatrix} 1 & -\rho_1 \\ 1 & -\rho_2 \end{bmatrix} \begin{bmatrix} m \\ v \end{bmatrix}$$

where ρ_1 and ρ_2 are the densities of the fluid, and m and v are the particle mass and volume, respectively. Solving directly, we get

$$\begin{bmatrix} m \\ v \end{bmatrix} = \frac{1}{\rho_2 - \rho_1} \begin{bmatrix} \rho_2 & -\rho_1 \\ 1 & -1 \end{bmatrix} \begin{bmatrix} m_{b_1} \\ m_{b_2} \end{bmatrix}$$

This leads us to the three identities for obtaining mass, volume and density from two buoyant mass measurements.

$$m = \frac{m_{b_1}\rho_2 - m_{b_2}\rho_1}{\rho_2 - \rho_1} \tag{1}$$

$$\rho = \frac{m_{b_1}\rho_2 - m_{b_2}\rho_1}{m_{b_1} - m_{b_2}} \tag{2}$$

$$v = \frac{m_{b_1} - m_{b_2}}{\rho_2 - \rho_1} \tag{3}$$

Importantly, mass and volume are linear combinations of the two buoyant mass measurements. Density, however is not. **Figure S3** shows the contour lines for both mass and density obtained from two buoyant mass measurements in H_2O and D_2O. Density is monotonically encoded in the angle of the two buoyant mass measurements, however this function is far from linear. For particles in the fourth quadrant (consisting of particles which sink in one fluid and float in the other), the transform between buoyant masses and density is relatively linear. However when the particle density is far beyond the density of either fluid (in the first or third quadrants), the gradient is extremely steep and so small errors in buoyant mass generate large errors in density.

To understand the error sources in our measurements, we first consider only the case of errors in buoyant mass estimation. We take these to be predominantly additive errors and estimate their magnitude by making repeated measurements on a single cell.

In calculating mass and volume, since they are linear combinations of buoyant masses, the errors are also transformed linearly. Hence, for a particle measured in H_2O ($\rho \approx 1.0$ g·cm^{-3}) and D_2O ($\rho \approx 1.1$ g·cm^{-3}) with buoyant mass errors with standard deviation σ_{m_b}, the standard deviation of the resulting mass estimate is $14.87\sigma_{m_b}$. For a particle measured in fluids of density ρ_1 and ρ_2, the standard deviation of $\hat{m}$ is

$$\sigma_{\hat{m}} = \frac{\sqrt{\rho_2^2 + \rho_1^2}}{(\rho_2 - \rho_1)} \sigma_{m_b}$$

Now we turn to the density estimator:

$$\hat{\rho} = \frac{m_{b_1}\rho_2 - m_{b_2}\rho_1}{m_{b_1} - m_{b_2}}$$

If we again assume that each buoyant mass measurement includes a random error ϵ_i (for the measurement made in fluid i), then we can rewrite the above by plugging in $m_{b_i} = m(1 - \frac{\rho_f}{\rho}) + \epsilon_i$

$$\begin{aligned} \hat{\rho} &= \frac{m(\rho_2 - \rho_1) + \rho_2\epsilon_1 - \rho_1\epsilon_2}{\frac{m}{\rho}(\rho_2 - \rho_1) + \epsilon_1 - \epsilon_2} \\ &= \rho \frac{m(\rho_2 - \rho_1) + \rho_2\epsilon_1 - \rho_1\epsilon_2}{m(\rho_2 - \rho_1) + \rho\epsilon_1 - \rho\epsilon_2} \end{aligned}$$

Here we see that the variance of the density estimator will depend on the true mass and density. We turn to Monte Carlo simulations to understand how a joint distribution over mass and density is affected by errors in buoyant mass. In particular, we take the true joint distribution to be constrained to only one possible density and then observe how the addition of noise to the buoyant mass measurements affects the observed joint distribution.

Evidence for complete fluid exchange

If the intracellular H_2O molecules were not being completely replaced by D_2O molecules, then we'd expect to measure a lower density (in the limit of no exchange occurring at all, we'd be measuring the total density of the particle, not the dry density). While we cannot be certain that 100% of the intracellular water has exchanged by the time we make the second measurement, we did verify that we do not see a statistically significant correlation between time spent in D_2O and dry density for *E. coli*. In yeast, of four replicate experiments, we only once saw a statistically significant correlation between dry density and time spent in D_2O, however the correlation explained only 5% of the variance in dry density, and suggested that the dry density was changing by 0.003 g cm^{-3} s^{-1}. This suggests that at a bare minimum, 2-3 seconds after immersing a cell in D_2O that the exchange process has reached an asymptote, which we think is likely to be near complete water exchange. This is consistent with previous findings (1).

Description of water-content measurement method

If a particle with a water volume of V_{water} is measured in a cell-impermeable fluid of density ρ_f, and that same particle is then placed in a cell-permeable fluid, also of density ρ_f and the buoyant mass is again measured, then we can write the buoyant masses obtained as:

$$m_{b_1} = m_{dry}\left(1 - \frac{\rho_f}{\rho_{dry}}\right) + m_{H2O}\left(1 - \frac{\rho_f}{\rho_{H2O}}\right)$$
$$m_{b_2} = m_{dry}\left(1 - \frac{\rho_f}{\rho_{dry}}\right)$$

Thus the water mass can be obtained as

$$m_{H2O} = \frac{m_{b_1} - m_{b_2}}{\left(1 - \frac{\rho_f}{\rho_{H2O}}\right)}$$

Necessity of single-cell measurements

While previous work in our lab and others (2,3) have used population means to estimate mean particle density, this method will not work for estimating dry density. The method used in these publications amounts to first measuring hundreds to thousands of particles in fluid 1, followed by measuring a similar number in fluid 2. The mean buoyant mass of each population is calculated, yielding μ_1 and μ_2. We then plug these in as m_{b_1} and m_{b_2} in supplementary equation 2. However, this measure relies on a reasonable degree of certainty in μ_1 and μ_2, which in turn depends on several factors:

- the actual dispersion of the population masses, σ_m
- the sample sizes used, n_1 and n_2
- the magnitude of the error in a single measurement, σ_ϵ

We're interested in the mean of the buoyant mass distribution in fluid i. Since the standard deviation of buoyant mass measurements in fluid i is given by $\sigma_i = \sqrt{\sigma_m^2(1 - \frac{\rho_i}{\rho})^2 + \sigma_\epsilon^2}$ and we assume measurements are independent and identically distributed, the standard error of $\hat{\mu}_i$ is $\sqrt{\frac{\sigma_m^2(1-\frac{\rho_i}{\rho})^2+\sigma_\epsilon^2}{n}}$. For very monodisperse particles (as were

typically measured in previous work (2,3)), this error is dominated by σ_ϵ, which is typically quite small. In the case of populations of cells, populations are often very heterogeneous (CV $\approx$ 33% for typical *E. coli* samples), and so σ_m dominates to such a degree that to achieve high precision in $\hat{\mu_i}$ requires tens to hundreds of thousands of cells, a number currently beyond the throughput of the SMR within a several-hour experiment. Making repeated single-cell measurements provides a much more accurate estimate of the true dry density because it avoids the problem of the population variance - each cell is measured in two fluids individually, and those measurements are paired together, allowing density determination without requiring population parameters.

References

1. Potma EO, de Boeij WP, van Haastert PJM, Wiersma, DA (2001) Real-time visualization of intracellular hydrodynamics in single living cells. *Proc Natl Acad Sci USA* 98, 1577-1582.

2. Godin M, Bryan AK, Burg TP, Babcock K, Manalis, SR (2007) Measuring the mass, density, and size of particles and cells using a suspended microchannel resonator. *Appl Phys Lett* 91, 123121.

3. Patel AR, Lau D, Liu J (2012) Quantification and characterization of micrometer and submicrometer subvisible particles in protein therapeutics by use of a suspended microchannel resonator. *Anal Chem* 84, 6833-6840.
